# Effects of Plantar Mechanical Stimulation on Anabolic and Catabolic Signaling in Rat Postural Muscle Under Short-Term Simulated Gravitational Unloading

**DOI:** 10.3389/fphys.2019.01252

**Published:** 2019-09-27

**Authors:** Sergey A. Tyganov, Ekaterina P. Mochalova, Svetlana P. Belova, Kristina A. Sharlo, Sergey V. Rozhkov, Natalia A. Vilchinskaya, Inna I. Paramonova, Timur M. Mirzoev, Boris S. Shenkman

**Affiliations:** Myology Laboratory, Institute of Biomedical Problems, Russian Academy of Sciences, Moscow, Russia

**Keywords:** skeletal muscle, hindlimb unloading, muscle atrophy, plantar mechanical stimulation, protein synthesis, protein degradation

## Abstract

It is known that plantar mechanical stimulation (PMS) is able to attenuate unloading-induced skeletal muscle atrophy and impaired muscle function. However, molecular mechanisms underlying the effect of PMS on skeletal muscle during unloading remain undefined. The aim of the study was to evaluate the effects of PMS on anabolic and catabolic signaling pathways in rat soleus at the early stages of mechanical unloading. Wistar rats were randomly assigned to ambulatory control, hindlimb suspension (HS) for 1 or 3 days, and HS for 1 or 3 days with PMS. The key anabolic and catabolic markers were assessed by western blotting and RT-PCR. Protein synthesis (PS) rate was estimated using SUnSET technique. PMS attenuated a 1-day HS-induced decrease in 4E-BP1, GSK-3β, and AMPK phosphorylation. PMS also partially prevented a decrease in PS, phosphorylation of GSK-3β, nNOS, and an increase in eEF2 phosphorylation after 3-day HS. PMS during 1- and 3-day HS prevented MuRF-1, but not MAFbx, upregulation but did not affect markers of ribosome biogenesis (18S + 28S rRNA, c-myc) as well as AKT phosphorylation. Thus, PMS during 3-day HS partially prevented a decrease in the global rate of PS in rat soleus muscle, which was accompanied by attenuation of MuRF-1 mRNA expression as well as changes in GSK-3β, nNOS, and eEF2 phosphorylation.

## Introduction

Skeletal muscles represent approximately 40% of human body mass. Therefore, the mass and composition of skeletal muscle are critical not only for muscle function itself but also for the state of the whole body. Skeletal muscle mass, structure, and metabolism can change due to mechanical loading and contractile activities. The extended periods of muscle inactivity caused by denervation (Midrio, 2006), immobilization (Udaka et al., 2011), and hindlimb unloading (Thomason and Booth, 1990; Globus and Morey-Holton, 2016) lead to a profound muscle atrophy, a complicated process associated with a significant reduction in muscle fiber cross-sectional area (CSA), protein content, muscle strength, and an increase in fatigability (Zhang et al., 2007). It is well-known that disuse muscle atrophy is muscle type-specific. Slow type postural muscles, such as soleus and adductor longus muscles, are more sensitive to microgravity/unloading conditions than fast twitch skeletal muscles (Roy et al., 1991; Fitts et al., 2001; Harrison et al., 2003; Kinoshita et al., 2006; Candasamy et al., 2014). At the molecular level, disuse-induced skeletal muscle atrophy results from decreased protein synthesis and increased protein degradation (Wang et al., 1991; Baldwin, 1996; Booth and Criswell, 1997; Bodine, 2013; Krivoi and Petrov, 2019).

Under conditions of Earth gravity, a number of biomechanical factors can influence the motor system of humans (as well as other mammals). Among these factors are *axial loading* (i.e., the effect of a body weight, which moves the center of mass toward a support surface leading to an inclination of the body axis) and *support reaction force*, which is directed toward body surface contacting with the substrate (Shenkman and Kozlovskaya, 2019). Mammals are able to perceive the effects of microgravity directly using the otolithic membrane of the inner ear, which detects changes in the position of the head as well as linear and centrifugal accelerations. At the same time, the musculoskeletal system perceives the effects of gravity indirectly. The sensory information from mechanical sensors of the soles of the feet significantly contributes to the maintenance of either posture or locomotion activity. The plantar contact is important in maintaining a vertical body position (Young et al., 1996) and it plays an important role in a microgravity-induced postural response (Layne and Spooner, 1990, 1992). The impact of biomechanical factors seen under real microgravity can be studied using “ground-based” models of simulated weightlessness such as hindlimb suspension (HS). Thus, in the present study, plantar mechanical stimulation was applied during simulated gravitational unloading (HS model).

The direct influence of support afferentation on human locomotor functions was first shown in the Soviet-Cuban joint experiment aboard the Soviet space vehicle that involved plantar mechanical stimulation (PMS) (Hernandez Corvo et al., 1983). Nemirovskaya and Shenkman (2002) have shown that constant passive support stimulation during hindlimb suspension can prevent a decrease in fiber CSA and muscle mass in rat soleus muscle (Nemirovskaya and Shenkman, 2002). It has also been shown that mechanical stimulation of the plantar foot surface was able to attenuate unloading-induced soleus muscle atrophy in rats (Kyparos et al., 2005). It was shown that both passive (without active inflation) and active (with active inflation) dynamic foot stimulation (DFS) resulted in protection of soleus type I myofibers from HS-induced atrophy. However, application of a DFS boot that had no plantar surface contact with the rat foot did not provide any significant protective effect. Based on this result, Kyparos et al. (2005) suggested that wearing the DFS boot is not associated with a “boot” loading effect or active recruitment of hindlimb musculature, rather is mediated *via* mechanical stimulation of the plantar surface. It appears that the effect of plantar mechanical stimulation is associated with stimulation of the cutaneous mechanoreceptors (Meissner corpuscles and Pacinian corpuscles) located in the plantar surface of the rat’s feet (Kyparos et al., 2005). Moreover, it seems that different modes of stimulation (active vs. passive) of plantar surface can activate distinct mechanoreceptors located in the upper epidermal or deeper dermal regions of plantar surface (Kyparos et al., 2005). De-Doncker et al. (2000) have demonstrated that mechanical stimulation of the cutaneous mechanoreceptors (10 min per day) of the soles of rat hindlimbs during 14-day unloading partially prevents soleus muscle atrophy (De-Doncker et al., 2000). In subsequent dry immersion studies, a modified plantar stimulation device was used allowing for a long-term series of stimulation. These studies have shown, in particular, that plantar stimulation under the conditions of dry immersion can facilitate the normal level of electrical activity and reflectory transversal stiffness in soleus muscle (for review, see Shenkman and Kozlovskaya, 2019). In human studies, PMS during simulated microgravity (7-day dry immersion model) was able to partially prevent an unloading-induced decease in fiber size, slow-to-fast fiber type shift, single fiber maximal tension, transverse stiffness as well as degradation of giant sarcomeric proteins (titin and nebulin) in soleus muscle (Moukhina et al., 2004; Ogneva et al., 2011; Shenkman and Kozlovskaya, 2019) Thus, evidence exists that plantar mechanical stimulation can be an effective countermeasure against unloading-induced atrophy of postural muscles. In addition, PMS can be used for attenuation of muscle atrophy in bedridden patients. However, molecular mechanisms underlying maintenance of structural and contractile characteristics of postural muscle with plantar stimulation under unloading conditions have not yet been studied. We hypothesized that plantar mechanical stimulation for 4 h per day during early unloading would attenuate a reduction in anabolic signaling and protein synthesis as well as preclude an increase in proteolytic markers in rat soleus muscle.

## Materials and Methods

### Ethical Approval

All procedures with the animals were approved by the Biomedicine Ethics Committee of the Institute of Biomedical Problems of the Russian Academy of Sciences/Physiology section of the Russian Bioethics Committee (protocol no. 414, 23.12.2015). All experiments were performed in strict accordance with the guidelines and recommendations as described by Grundy (2015). All efforts were made to minimize the animals’ pain and suffering. Animals were housed in a temperature-controlled room on a 12: 12-h light-dark cycle with food pellets and water provided ad libitum. Thirty two 3-month-old Wistar male rats were obtained from the certified Nursery for laboratory animals of the Institute of Bioorganic Chemistry of the Russian Academy of Sciences (Pushchino, Moscow region). Prior to all surgical procedures, the animals were anesthetized with an intraperitoneal injection of tribromoethanol (240 mg/kg). The depth of anesthesia was evaluated by testing the pedal withdrawal reflex (toe and foot pad pinch).

### Hindlimb Suspension and Animals

Male Wistar rats weighing 225 ± 10 g were obtained from the certified nursery for laboratory animals of the Institute of Bioorganic Chemistry of the Russian Academy of Sciences (Pushchino, Moscow region). Mechanical unloading was simulated using a standard hindlimb suspension (HS) model (Morey-Holton and Globus, 2002). Two consecutive experiments were performed. In *Experiment 1*, 21 rats were randomly assigned to the following three groups (*n* = 7/group): (1) vivarium cage control (C); (2) hindlimb suspension for 1 day (1HS); and (3) HS for 1 day plus plantar mechanical stimulation (PMS) for 4 h per day (PMS). In *Experiment 2*, 21 rats were randomly assigned to the following groups (*n* = 7/group): (1) vivarium cage control (C); (2) hindlimb suspension for 3 days (3HS); and (3) HS for 3 days plus PMS for 4 h per day (PMS).

Serum corticosterone level has been evaluated using Corticosterone EIA kit (immunodiagnostic systems, AC-14F1).

### Plantar Mechanical Stimulation

PMS apparatus was attached to the hindlimbs without removing the animals from HS position to stimulate the cutaneous mechanosensory receptors in the soles of the rat’s foot in conscious animals. PMS apparatus is the plastic custom-built boot with a movable platform inside that allows regulating pressure and frequency of the sole stimulation. PMS apparatus was attached to the animal foot above the ankles using the adhesive patch. Pressure was applied to the foot by movable platform contacting with the sole of the foot using an electronically controlled custom-built air pump attached to a hose. Each sole was stimulated with a frequency of 1-s inflation/1-s deflation with a total 20 min followed by 10 min rest (Kyparos et al., 2005). This cycle was repeated eight times within 4 h each day of HS. Apparatus was removed after completion of all cycles. To sufficiently stimulate cutaneous mechanosensory receptors within the sole of the animal foot, pressure that exceeds their mechanical threshold (i.e., >8 mN) needs to be applied (Leem et al., 1993). In a previous report by Kyparos et al. (2005), the pressure required to stimulate the entire plantar surface was calculated as 13.9 mN/mm^2^ (104 mmHg) (Kyparos et al., 2005). This pressure was set in the current experiment.

### Surface Sensing of Translation Technique for Measuring the Rate of Protein Synthesis

Surface sensing of translation (SUnSET) is a nonradioactive technique that allows measuring the rate of protein synthesis (PS) in skeletal muscle. This technique involves the use of the antibiotic puromycin (a structural analogue of tyrosyl-tRNA), and anti-puromycin antibodies to detect the amount of puromycin incorporation into nascent peptide chains. It was shown that when puromycin is used at low concentrations (40 nmol/g), the accumulation of puromycin-conjugated peptides accurately reflects the rate of protein synthesis (Goodman et al., 2011). SUnSET technique uses standard Western blotting and immunohistochemical technologies to visualize and quantify the rates of protein synthesis (Schmidt et al., 2009). For measurements of protein synthesis, rats were given an intraperitoneal injection of 0.04 μmol/g puromycin hydrochloride (Enzo Life Sciences, NY, USA) dissolved in PBS. At exactly 30 min after injection, muscle tissue was extracted and frozen in liquid nitrogen for WB analysis.

### Western Blot Analysis

The skeletal muscle tissue (30 mg) was homogenized in the ice-cold lysis buffer: 50 mM Tris (pH 7.4), 150 mM NaCl, 1% Nonidet P-40, 0.5% sodium deoxycholate, 0.1% SDS, 0.004% sodium azide, and 5 mM EDTA, supplemented with 1 mM DTT, 1 mM PMSF, 10 μg/ml leupeptin, 5 μl/ml pepstatin, and 1% aprotinin (Sigma-Aldrich, MO, USA), mammalian protease inhibitor cocktail (Amresco, Solon, OH, USA), and phosphatase inhibitor cocktail B (Santa Cruz Biotechnology, CA, USA). The total protein concentration of the lysates was determined by incubation for 20 min at 4°C and centrifugation for 10 min at 12,000 *g*. The samples were diluted in Laemmli buffer. Cytoplasmic and nuclear protein fractions from skeletal muscle tissue were separated and isolated using NE-PER Nuclear and Cytoplasmic Extraction kit (Thermo Scientific, USA) following the manufacturer instructions. The protein content of the supernatants was quantified using an assay based on a modification of the Bradford protocol (Bradford, 1976). Bovine serum albumin was used as a standard. The total amount of 10–45 μg protein was subjected to SDS-PAGE (Laemmli, 1970), and then were transferred to nitrocellulose membrane (Bio-Rad Laboratories, CA, USA). Then, to verify equal loading of protein in all lanes, the nitrocellulose membrane was dyed by Ponceau S. The membranes were blocked for 1 h at room temperature with the blocking buffer (4% nonfat milk powder, TBS, pH 7.4, and 0.1% Tween 20) and incubated overnight at 4°C with primary antibodies (diluted in TBS-T) against p-p70S6K (Thr 389) (1:2,000, Santa Cruz Biotechnology, USA, sc-11759) and p70s6k (1:1,000, Cell Signaling Technology, USA, #9202), p-4E-BP1 (Thr37/46) (1:1,000, Cell Signaling Technology, USA, #2855), and 4E-BP-1 (1:1,000, Cell Signaling Technology, USA, #9452), p-GSK-3β (Ser 9) (1:1,000, Cell Signaling Technology, USA, #9322), and GSK-3β (1:1,000, Cell Signaling Technology, USA, #12456), p-eEF2 Thr56 (1:1,000, Cell Signaling, USA, #2331) and t-eEF2 (1:1,000, Cell Signaling Technology, USA, #2332), p-AKT (Ser473) (1:1,000, Cell Signaling Technology, USA, #4058) and AKT (1:1,000, Cell Signaling Technology, USA, #9272), puromycin (1:3,000, Kerafast Inc., Boston, USA, EQ0001), GAPDH (1:10,000, Applied Biological Materials Inc., Richmond, British Columbia, Canada, no. G041), p-FOXO3 (Ser253) (1:1,000, Cell Signaling Technology, USA, #9466) and FOXO3 (1:1,000, Cell Signaling Technology, USA, #2497), p-PKD (Ser916) (1:500, Cell Signaling Technology, USA, #2051) and PKD (1:1,000, Cell Signaling Technology, USA, #2052), p-AMPK (Thr172) (1:1,000, Cell Signaling Technology, USA, #2531) and AMPK (1:1,000, Cell Signaling Technology, USA, #2532), HDAC5 (1:4,000, abcam, USA, ab1439), Lamin B1 (1:1,000, abcam, USA, ab16048). Three 10-min washes with TBS-T were then performed. After that, the membranes were incubated for 1 h at room temperature with horseradish peroxidase-conjugated secondary antibodies to rabbit (1:30,000, Jackson Immuno Research, USA, #111-035-003) or mouse (diluted 1:20,000; Bio-Rad Laboratories, CA, USA, # 1706516) immunoglobulins. The membranes were then washed again in TBS-T three times for 10 min and incubated in Immun-Star HRP Chemiluminescent system (Bio-Rad Laboratories, Hercules, CA, USA). The protein bands were quantified using C-DiGit Blot Scanner (LI-COR Biotechnology, USA) and Image Studio Digits software. Following image capture of phosphorylated proteins, membranes were stripped of the phosphospecific antibodies, using RestoreTM Western Blot Stripping Buffer (Thermo Scientific, USA), for 30 min at 37°C after which the membranes were re-probed with primary antibodies for each respective total protein. The signal from the phosphoprotein was normalized to the total protein. For protein synthesis detection, the measurements of the chemiluminescent signals were performed by determining the density of each whole lane with the entire molecular weight range of puromycin-labeled peptides. Each gel contained samples from the all groups. Protein samples were run at least in duplicate on the same gel. The representative blots are of the same samples (phospho and total). GAPDH content was used as loading control.

### RNA Preparation and Electrophoresis

The samples of muscle tissue were sliced using cryostat (Leica, Germany) and weighed on electronic laboratory balance. Total RNA was extracted from frozen soleus muscle samples using RNeasy Micro Kit (Qiagen, Germany) according to the manufacturer’s protocol. RNA concentration was analyzed at 260 nm. RNA quality of purification was evaluated according to 260/280 and 260/230 ratios. The electrophoresis was carried out in 1.2% agarose gel with ethidium bromide staining in TBE buffer. The total mRNA sample value for electrophoretic gel was evaluated by normalization of the sum value of RNA extracted from the tissue sample to a tissue sample weight. All the samples were mixed with equal value of denaturing buffer (Thermo Scientific) and heated for 70°C for 10 min according to manufacturer’s protocol. RiboRuler markers (Thermo Scientific) were used for RNA molecular weight analysis. The results of electrophoresis were analyzed by Gel Doc EZ Imager (Biorad) and Image Studio Digits v. 4.0. software. The rest of RNA solutions were stored at −85°C and until further RT-PCR procedures. The RNA integrity was assessed by evaluating 28S/18S ratio.

### Gene Expression Analysis

Expression of c-myc, eEF-2 k, PGC-1alfa, MAFbx, MuRF-1 was analyzed using RT-PCR. The following primers were used for PCR:

**Table 1:** 

c-myc-F	ttgatggggatgaccctgac
c-myc-R	ctcgcccaaatcctgtacct
PGC-1α-F	ctgccattgttaagaccgagaa
PGC-1α-R	tggcctcgttgtcagtggtc
MAFbx-F	ctacgatgttgcagccaaga
MAFbx-R	ggcagtcgagaagtccagtc
MuRF-1-F	gccaatttggtgctttttgt
MuRF-1-R	aaattcagtcctctccccgt
eEF-2k-F	agaagctggtgacaggcagt
eEF-2k-R	gggttcttgtccagtccaaa
SUMO-F	agctcccttaacattgccct
SUMO-R	aactgcagggccattgaaag

Reverse transcription was performed by incubation of 0.5 μg of RNA, random hexamers d(N)6, dNTPs, RNase inhibitor, and MMLV reverse transcriptase for 60 min at 42°C. The samples to be compared were run under similar conditions (template amounts, duration of PCR cycles). The annealing temperature was based on the PCR primers’ optimal annealing temperature. The amplification was real-time monitored using SYBR Green I dye and the iQ5 Multicolor Real-Time PCR Detection System (Bio-Rad Laboratories, USA). To confirm the amplification specificity, the PCR products from each primer pair were subjected to a melting curve analysis and sequencing of the products was provided at least once. Relative quantification was performed based on the threshold cycle (CT value) for each of the PCR samples (Livak and Schmittgen, 2001). SUMO1 was tested and chosen for the normalization of all quantitative PCR analysis experiments in the current study.

### Statistical Analysis

All WB data are expressed as means ± SEM, all RT-PCR and rRNA data are expressed as median and interquartile range (0.25–0.75) ± the minimum and the maximum. Since the data were not normally distributed, a nonparametric Kruskal-Wallis test was used, and *post hoc* analysis was performed using a Dunn’s multiple range test with *p* < 0.05 designated as statistically significant.

## Results

There was no significant difference between the groups in terms of body weight. Soleus weight to body weight ratio did not significantly differ between ambulatory controls and hindlimb suspended rats with and without PMS (data not shown). In order to find out if the rats were stressed under short-term HS, serum corticosterone level has been evaluated in control and 1-HS rats with or without PMS. No significant differences in corticosterone concentration were observed between experimental groups: Сontrol – 886.7 ± 29.7 ng/ml; 1HS – 847.1 ± 93.5 ng/ml; PMS – 841.3 ± 81.1 ng/ml (data presented as means ± SEM).

### Anabolic Signaling Markers

**Figure 1:**
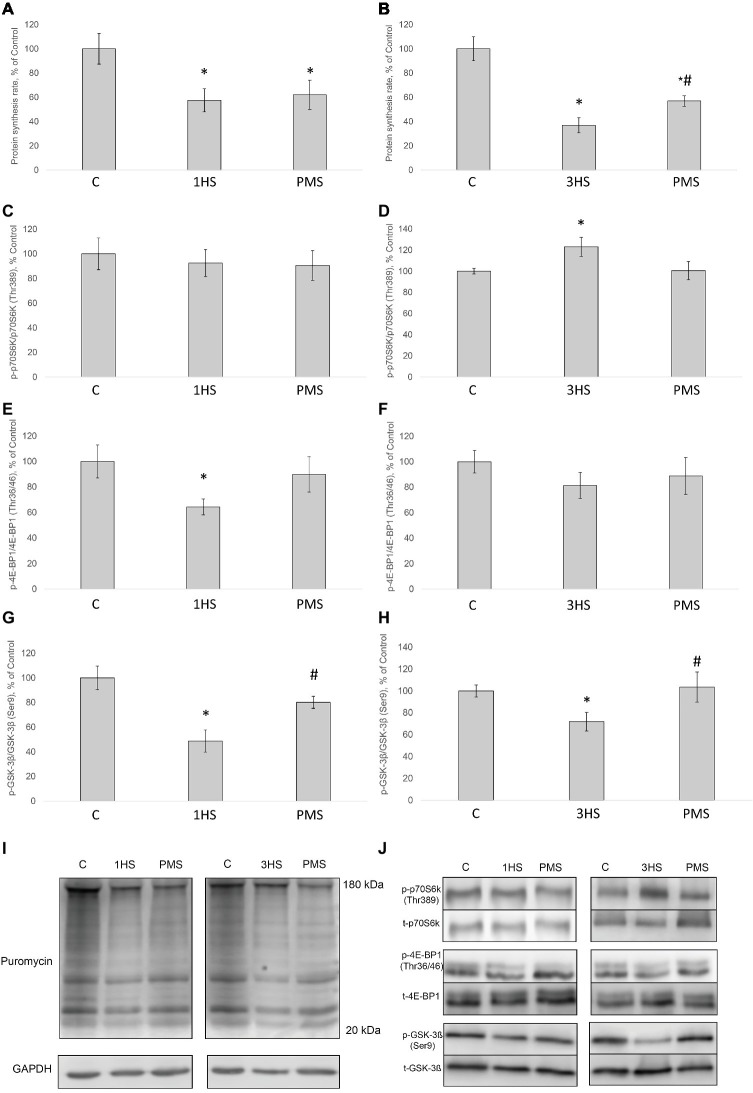
The rate of protein synthesis and phosphorylation status of the signaling proteins in the rat soleus muscle after hindlimb suspension and plantar mechanical stimulation. Quantification of the puromycin-labeled peptides after 1-day HS **(A)** and 3-day HS **(B)**. Quantification of phospho-p70s6k/total p70s6k ratio after 1-day HS **(C)** and 3-day HS **(D)**. Quantification of phospho-4E-BP1/total 4E-BP1 ratio after 1-day HS **(E)** and 3-day HS **(F)**. Quantification of phospho-GSK-3β/total GSK-3β ratio after 1-day HS **(G)** and 3-day HS **(H)**. Representative immunoblot for the PS measure **(I)**. Representative immunoblots for the studied proteins in the rat soleus muscle **(J)**. C, control group; 1HS, 1-day hindlimb suspension; 3HS, 3-day hindlimb suspension; PMS, 1- or 3-day HS plus plantar mechanical stimulation. All data expressed relative (%) to control. ^*^Significant difference vs. C, *p* < 0.05; ^#^significant difference vs. HS. All values are means ± SE, *n* = 7/group.

**Figure 2:**
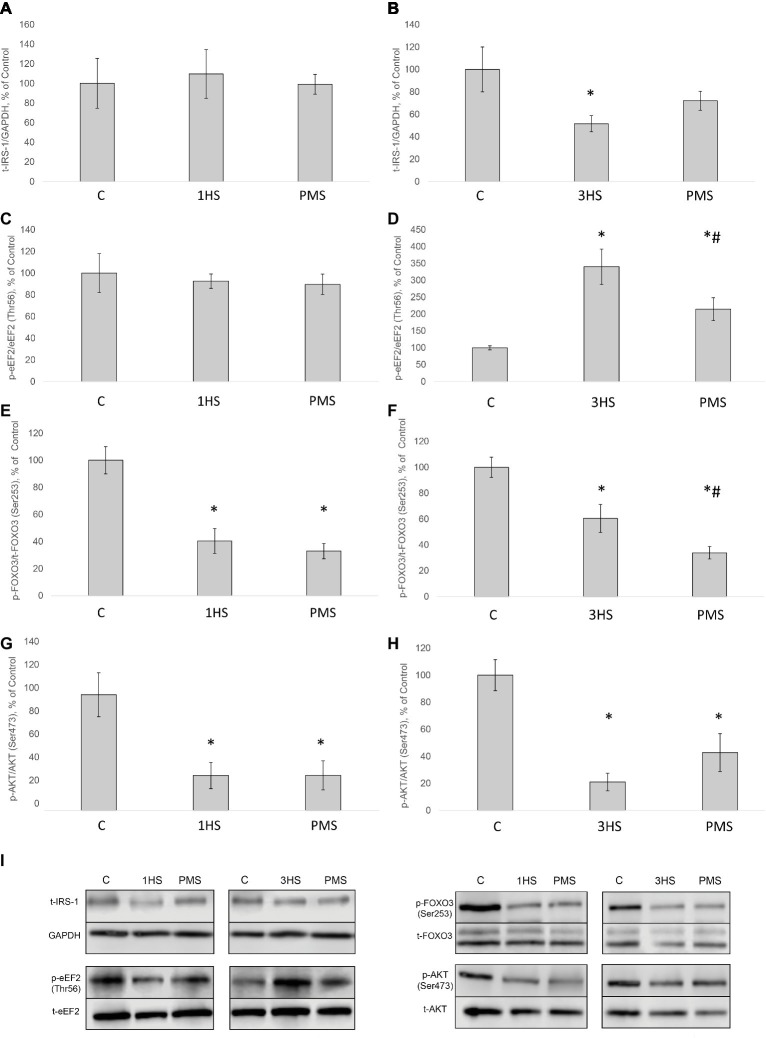
Phosphorylation status of the signaling proteins in the rat soleus muscle after hindlimb suspension and plantar mechanical stimulation. Quantification of total IRS-1/GAPDH ratio after 1-day HS **(A)** and 3-day HS **(B)**. Quantification of phospho-eEF2/total eEF2 ratio after 1-day HS **(C)** and 3-day HS **(D)**. Quantification of phospho-FOXO3/total FOXO3 ratio after 1-day HS **(E)** and 3-day HS **(F)**. Quantification of phospho-AKT/total AKT after 1-day HS **(G)** and 3-day HS **(H)**. Representative immunoblots for the studied proteins in the rat soleus muscle **(I)**. C, control group; 1HS, 1-day hindlimb suspension; 3HS, 3-day hindlimb suspension; PMS, 1- or 3-day HS plus plantar mechanical stimulation. All data expressed relative (%) to control. ^*^Significant difference vs. C, *p* < 0.05; ^#^significant difference vs. HS. All values are means ± SE, *n* = 7/group.

**Figure 3:**
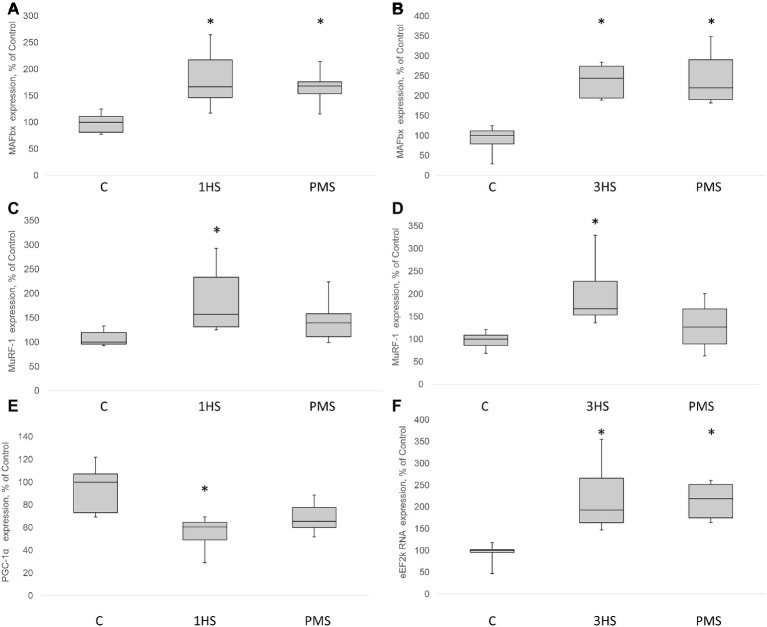
MAFbx, MuRF-1, PGC-1α and eEF2k mRNA content in the rat soleus muscle after hindlimb suspension and plantar mechanical stimulation. MAFbx mRNA expression after 1-day HS **(A)** and 3-day HS **(B)**. MuRF-1 mRNA expression after 1-day HS **(C)** and 3-day HS **(D)**. PGC-1α mRNA expression after 3-day HS **(E)**. eEF2k mRNA expression after 3-day HS **(F)**. C, control group; 1HS, 1-day hindlimb suspension; 3HS, 3-day hindlimb suspension; PMS, 1- or 3-day HS plus plantar mechanical stimulation. All data expressed relative (%) to control. Values are medians and interquartile range (0.25–0.75) ± the minimum/maximum. ^*^Significant difference vs. C, *p* < 0.05, *n* = 7/group.

SUnSET measurements revealed that PS was significantly decreased by 42% after the first day of HS compared to control group (Figures 1A,I). PMS did not prevent this decrease. Three-day HS resulted in a subsequent loss in PS compared to control values. However, PMS partly attenuated this 3-day unloading-induced decrease in PS (Figures 1B,I). IGF-1/AKT/mTORC1 is one of the key signaling pathways that plays a major role in the regulation of PS (Schiaffino and Mammucari, 2011). Ribosomal protein S6 kinase p70 (p70S6k) and eukaryotic translation initiation factor 4E-binding protein 1 (4E-BP1) are well-known downstream targets of mammalian/mechanistic target of rapamycin complex 1 (mTORC1), a key protein complex involved in the control of PS. In the present study, p70S6k phosphorylation was not altered after 1 day of HS (Figures 1C,J). Nevertheless, 3-day HS led to a 23% increase in p70S6k phosphorylation (*p* < 0.05) compared with control (Figures 1D,J). PMS did not alter p70S6k phosphorylation after 1-day HS but prevented an increase in p70S6k phosphorylation following 3-day HS (Figures 1C,D,J). 4E-BP1 phosphorylation after 1- and 3-day HS did not differ from the control values. PMS during HS did not affect 4E-BP1 phosphorylation as well (Figures 1E,F,J). Glycogen synthase kinase 3 beta (GSK-3β) can regulate mRNA translation initiation *via* eukaryotic translation initiation factor 2B (eIF2B) (Welsh et al., 1998). GSK-3β phosphorylation was significantly decreased by 52% (*p* < 0.05) after 1-day HS and by 36% (p < 0.05) after 3-day HS compared to the control group. PMS was able to prevent this decrease in GSK-3β (Ser9) phosphorylation (Figures 1G,H,J). GSK-3β is a downstream target of protein kinase B (AKT) which can inhibit GSK-3β activity *via* Ser9 phosphorylation (Jefferson et al., 1999; Leger et al., 2006). Phospho-AKT content was significantly decreased after 1- and 3-day HS, and PMS did not affect this decline (Figures 2G–I). Eukaryotic elongation factor 2 (eEF2) is a key component of protein translation machinery. Hyperphosphorylation of eEF2 leads to preventing its binding to the ribosome thereby impairing elongation rate (Ryazanov and Davydova, 1989; Redpath et al., 1993). eEF2 phosphorylation did not differ from the control group after 1-day HS. However, eEF2 phosphorylation was significantly increased after 3-day HS and PMS partly attenuated this effect (Figures 2C,D,I). Also, eEF2 kinase mRNA expression was significantly increased after 3-day HS with or without PMS compared to the control group (Figure 3F).

**Figure 4:**
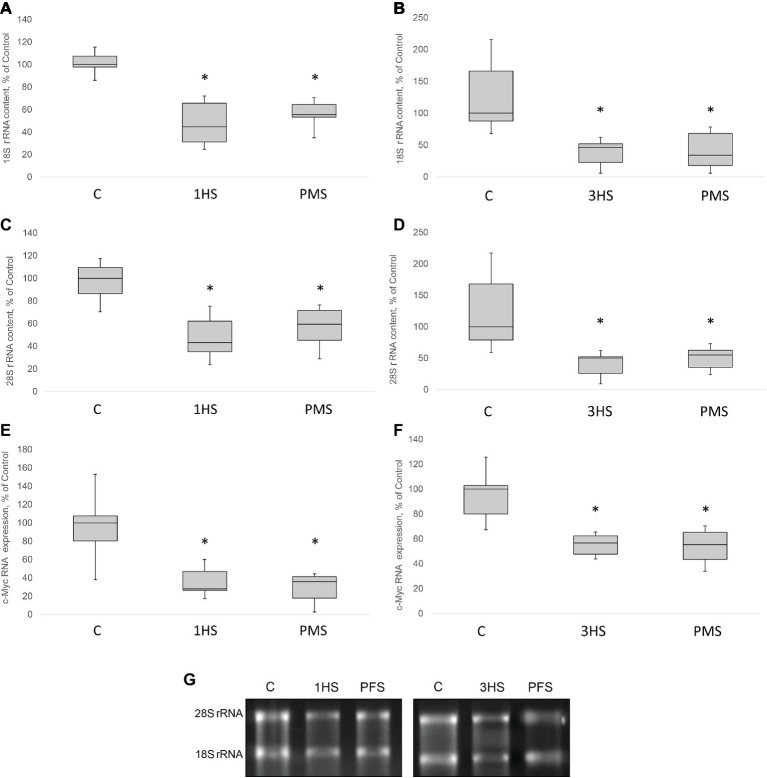
18S, 28S rRNA content and relative c-Myc mRNA content in the rat soleus muscle after hindlimb suspension and plantar mechanical stimulation. 18S rRNA content after 1-day HS **(A)** and 3-day HS **(B)**. 28S rRNA content after 1-day HS **(C)** and 3-day HS **(D)**. c-Myc mRNA expression after 1-day HS **(E)** and 3-day HS **(F)**. Representative rRNA electrophoresis **(G)**. C, control group; 1HS, 1-day hindlimb suspension; 3HS, 3-day hindlimb suspension; PMS, 1- or 3-day HS plus plantar mechanical stimulation. All data expressed relative (%) to control. Values are medians and interquartile range (0.25–0.75) ± the minimum/maximum. ^*^Significant difference vs. C, *p* < 0.05, *n* = 7/group.

In skeletal muscle, the rate of protein synthesis per ribosome (translational efficiency) and the amount of ribosomes per unit tissue (translational capacity) can be affected by both hypertrophic and atrophic stimuli (Baar and Esser, 1999; De Boer et al., 2007; McCarthy and Esser, 2007). There is an emerging recognition that the initial decrease in translational efficiency in response to gravitational unloading is required for the subsequent decrease in translational capacity that ultimately leads to the reduction in PS rate and skeletal muscle atrophy. 18S and 28S rRNAs are the parts of large and small subunits of eukaryotic ribosome that serve as markers of ribosome biogenesis (Proud, 2013; Thomson et al., 2013). RNA electrophoresis indicated that 18S rRNA significantly decreased in muscle tissue by 57% after 1-day HS and by 54% after 3-day HS (Figures 4A,B,G). In addition, a significant decrease in 28S rRNA content by 50% after 1-day HS and 56% after 3-day HS occurred (Figures 4C,D,G). A similar significant decline in both 18S and 28S rRNA was observed in unloaded groups with PMS (Figures 4A–D). The Wnt/β-catenin/c-myc pathway has been shown to be critical for skeletal muscle development and ribosome biogenesis. c-myc is a transcription factor that is known to regulate RNA polymerase 1 activity and the transcription of several large and small ribosome subunit genes, while c-myc inactivation results in a reduction in ribosomal protein gene expression (Coller et al., 2000; Chaillou et al., 2014). c-myc mRNA expression was significantly decreased in both unloaded groups as compared to the control group and application of PMS during unloading was not able to prevent such a decrease (Figures 4E,F).

### Catabolic Signaling Markers

**Figure 5:**
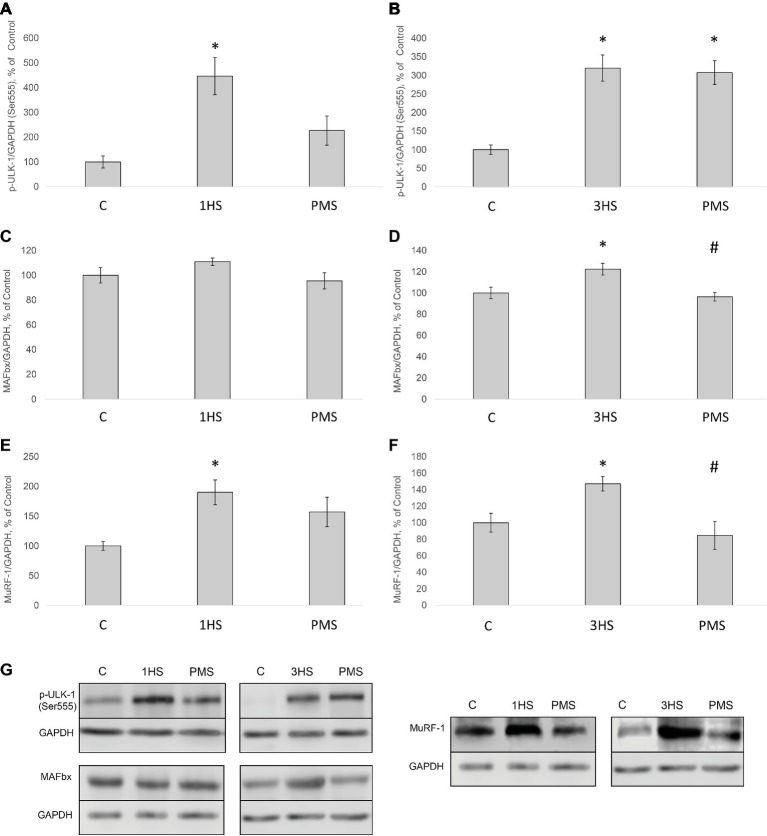
Phosphorylation status of the signaling proteins in the rat soleus muscle after hindlimb suspension and plantar mechanical stimulation. Quantification of phospho-ULK-1/total ULK-1 ratio after 1-day HS **(A)** and 3-day HS **(B)**. Quantification of MAFbx/GAPDH after 1-day HS **(C)** and 3-day HS **(D)**. Quantification of MuRF-1/GAPDH after 1-day HS **(E)** and 3-day HS **(F)**. Representative immunoblots for the studied proteins in the rat soleus muscle **(G)**. C, control group; 1HS, 1-day hindlimb suspension; 3HS, 3-day hindlimb suspension; PMS, 1- or 3-day HS plus plantar mechanical stimulation. All data expressed relative (%) to control. ^*^Significant difference vs. C, *p* < 0.05; ^#^significant difference vs. HS. All values are means ± SE, *n* = 7/group.

Insulin receptor substrate 1 (IRS-1) plays a key role in transmitting signals from insulin-like growth factor-1 (IGF-1) receptors to PI3K/Akt intracellular pathway (Haddad et al., 2006). There were no significant differences in IRS-1 content after 1-day HS. However, IRS-1 content significantly decreased by 49% after 3-day HS. PMS was able to partially prevent this IRS-1 degradation (Figures 2A,B,I). Muscle RING finger 1 (MuRF1) and muscle atrophy F-box (MAFbx)/atrogin-1 were identified as two muscle-specific E3 ubiquitin ligases (markers of the ubiquitin-proteasome system) that increase their expression in skeletal muscle under atrophy-inducing conditions (Bodine and Baehr, 2014). MuRF-1 and MAFbx expression was significantly increased after 3-day HS by 62 and 144%, respectively. PMS prevented this increase only for MuRF-1 (Figures 3A–D). The protein content of MuRF-1 increased after 1- and 3-day HS by 90 and 47%, respectively, and PMS also prevented this increase (Figures 5E–G). MAFbx protein content increased after 3-day HS by 22% and was prevented by PMS as well (Figures 5C,D,G). Forkhead box O3 (FoxO3) is considered to be a key transcriptional factor regulating MuRF-1 and MAFbx expression. It was also shown, that FoxO3 activation causes a dramatic decrease in muscle fiber size (Mirzoev et al., 2017). Akt has been shown to phosphorylate FoxO3 on Thr32, Ser253, and Ser315 residues, leading to its exclusion from the nucleus and cytoplasmic retention (Lin and Liu, 2019). In our experiment, phosphorylated FoxO3 content in rat soleus muscle decreased after 1- and 3-day HS with or without PMS (Figures 2E,F,I).

Peroxisome proliferator-activated receptor gamma co-activator 1-alpha (PGC-1α) plays a key role in the maintenance of glucose, lipid, and energy homeostasis in muscle and other tissues. PGC-1α can function as factor opposing the effects of FoxO3 on muscle mass (Sandri et al., 2006; Olesen et al., 2010). In the present study, 3-day HS induced a significant reduction in PGC-1α mRNA expression; however application of PMS partly attenuated an unloading-induced decrease in PGC-1α expression (Figure 3E). Another protein degradation system that can be activated under a number of muscle wasting conditions is the autophagy–lysosome system (Sandri, 2013). There is evidence that autophagy markers (ULK1, LC3, p62, and beclin1) can be upregulated in skeletal muscle under disuse conditions (Brocca et al., 2012; Egawa et al., 2015; Moller et al., 2019). In the present study, the content of phospho-ULK1 was significantly increased after 1- and 3-day HS for 346 and 220% respectively. Plantar stimulation prevented this effect only after 1-day HS (Figures 5A,B,G).

### Impact of AMPK on Anabolic Processes

**Figure 6:**
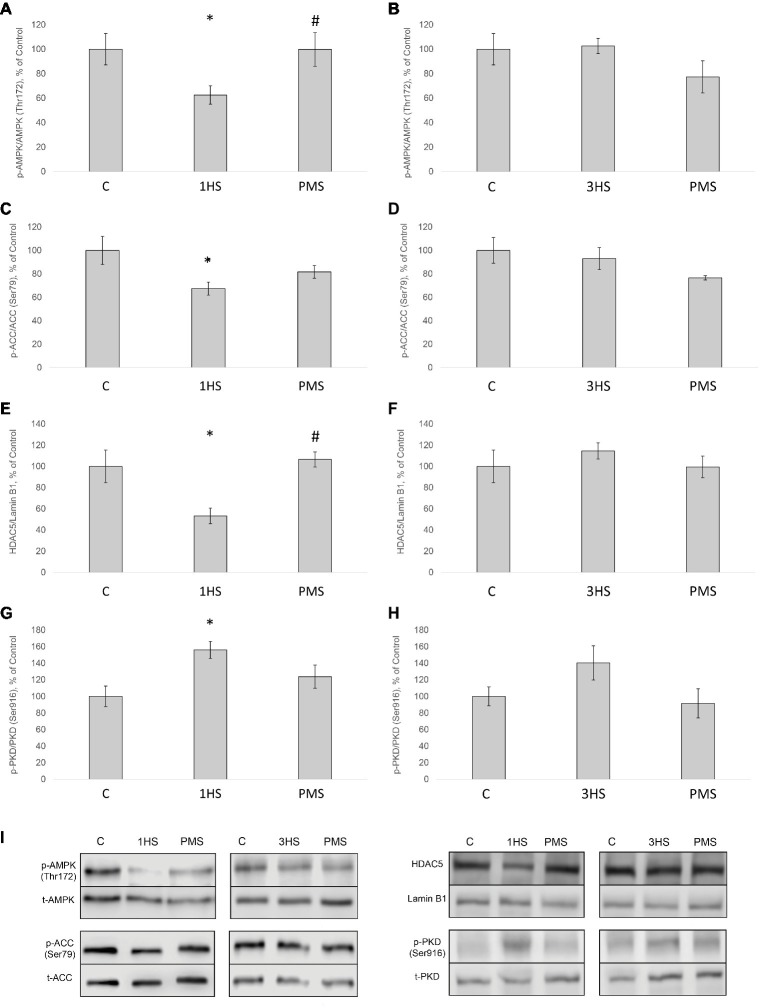
Phosphorylation status of the signaling proteins in the rat soleus muscle after hindlimb suspension and plantar mechanical stimulation. Quantification of phospho-AMPK/total AMPK ratio after 1-day HS **(A)** and 3-day HS **(B)**. Quantification of phospho-ACC/total ACC ratio after 1-day HS **(C)** and 3-day HS **(D)**. Quantification of HDAC5/Lamin B1 ratio after 1-day HS **(E)** and 3-day HS **(F)**. Quantification of phospho-PKD/total PKD after 1-day HS **(G)** and 3-day HS **(H)**. Representative immunoblots for the studied proteins in the rat soleus muscle **(I)**. C, control group; 1HS, 1-day hindlimb suspension; 3HS, 3-day hindlimb suspension; PMS, 1- or 3-day HS plus plantar mechanical stimulation. All data expressed relative (%) to control. ^*^Significant difference vs. C; *p* < 0.05; ^#^significant difference vs. HS. All values are means ± SE, *n* = 7/group.

It is known that AMP-activated protein kinase (АМРK) can suppress anabolic processes *via* phosphorylation of TSC2 and raptor, thereby inhibiting mTORC1 and protein synthesis (Armstrong and Phelps, 1984; Bolster et al., 2002; Mounier et al., 2009; Wood et al., 2014; Moller et al., 2015). In the present study, AMPK (Thr172) phosphorylation in cytoplasmic protein fraction significantly declined by 38% after 1-day HS compared to the control group. PMS was able to prevent this decrease (Figures 6A,I). After 3-day HS, no significant differences in AMPK phosphorylation in both HS and PMS groups were detected as compared to the control group (Figures 6B,I). A similar pattern of phosphorylation was observed for acetyl-CoA carboxylase (ACC), which is considered to be a marker of AMPK activity (Figures 6C,D,I; Brownsey et al., 2006). AMPK is also known to be involved in the regulation of class IIa histone deacetylases (HDACs) (McGee and Hargreaves, 2010). In mammals, the class IIa HDACs comprise a family of four functionally overlapping members: HDAC4, HDAC5, HDAC7, and HDAC9 (Haberland et al., 2009). After 1-day HS HDAC5 content in nuclear fraction significantly decreased by 47%. This effect was prevented by PMS (Figures 6E,I). However, after 3-day HS, there was no significant difference in the nuclear HDAC5 content in both HS and HS + PMS groups compared to the control group (Figures 6F,I). HDAC5 can be regulated by protein kinase D (PKD) as well (Rockl et al., 2007; McGee et al., 2014). Following 1-day HS, PKD (Ser916) phosphorylation was significantly increased by 56%. Application of PMS during 1-day unloading was able to attenuate increased PKD phosphorylation (Figures 6G–I).

## Discussion

### Anabolic Signaling Markers

Loughna et al. (1986) were the first, who have shown a significant decrease in the fractional rate of PS in rat soleus muscle following 3-day HS. These authors have also demonstrated that protein synthetic rate is enhanced when inactive soleus muscle is permanently stretched (Loughna et al., 1986). Using puromycin-based SUnSET technique, in our laboratory, it has been recently shown that the rate of PS in rat soleus muscle is significantly reduced after 3- and 7-day hindlimb unloading (Mirzoev et al., 2016). In the present study, 24 h of HS induced a significant decrease in the rate of PS and further decline following 3 days of HS. The reduction in the rate of PS was partially prevented by PMS during 3 days of unloading. Alterations in the rate of PS could be associated with the changes in phosphorylation status of the key signaling molecules involved in the regulation of translational efficiency (Mirzoev et al., 2016). It has been previously shown that negative GSK-3β Ser9 phosphorylation in rat soleus is observed at the early stages of unloading (Dupont et al., 2011; Mirzoev et al., 2016). In the present study, a decrease in the level of negative GSK-3β phosphorylation was observed after 3 days of HS and was completely prevented by PMS. At the same time phosphorylation of Akt, which lies upstream of GSK-3β, significantly decreased after mechanical unloading and was not affected by PMS. However, the level of GSK-3β phosphorylation can also depend on cGMP-dependent kinases, which are activated by nitric oxide (NO) (Drenning et al., 2008). Previously, it was shown in our laboratory that HS can lead to a significant reduction in NO content in rat soleus muscle (Von Walden et al., 2012). In humans, a significant decrease in the content of neuronal NO-synthase (nNOS) was found in soleus muscle after 7 days of unloading (dry immersion), which was prevented by the use of PMS (Moukhina et al., 2004). It is possible that the maintenance of nNOS content allowed keeping the content of NO at the level of intact control leading to the prevention of a decrease in GSK-3β phosphorylation. Phosphorylation of eEF2, that controls translation elongation, can occur *via* Ca-calmodulin-dependent activation of eEF2-kinase. We have previously shown that 14-day HS results in a significant increase in eEF2 (Thr56) phosphorylation in rat soleus muscle (Lomonosova et al., 2017). In the present study, 3 days of HS induced a significant increase in the level of eEF2 (Thr56) phosphorylation that was partially attenuated by PMS. Thus, PMS during 3-day unloading allowed for partial prevention of the inhibitory effect of GSK-3β and eEF2 on translational efficiency in rat soleus muscle.

Ribosome biogenesis is the primary determinant of translational capacity, but its regulation in skeletal muscle during mechanical unloading is poorly studied. It has been previously shown that hindlimb unloading leads to a significant decrease in the expression of ribosomal RNA (rRNA) in rat soleus muscle (Bajotto et al., 2011; Mirzoev et al., 2016). In the present study, we found a profound decrease in the amount of 18S + 28S rRNAs as well as c-myc in rat soleus after 1- and 3 days of HS. The use of support stimulation did not affect the reduction of these parameters, as well as the expression of c-Myc, one of the main regulators of rRNA expression. Thus, PMS was insufficient for maintaining a normal level of ribosome biogenesis at the early stage of unloading.

There is contradictory evidence concerning p70S6K phosphorylation status in rodent postural muscles during mechanical unloading. Sugiura et al. (2005) reported about the lack of changes in phospho-p70S6K content in rat soleus following 10 days of HS (Sugiura et al., 2005). At the same time, it has been shown a significant decrease in p70S6K phosphorylation after 7 days of unloading (Bajotto et al., 2011; Dupont et al., 2011). However, 3 days of hindlimb immobilization induced a significant increase in the level of p70S6K phosphorylation in mouse soleus muscle (Bertsch et al., 2011). This increased p70S6K phosphorylation during early unloading is in accordance with several recent reports (Mirzoev et al., 2016; Uchida et al., 2018). In the present study, 3 days of HS resulted in a significant increase in the level of p70S6K phosphorylation, which was completely prevented by PMS. We have previously suggested that an increase in p70S6K (Thr389) phosphorylation following 1-day HS could be associated with a decrease in AMPK (Thr172) phosphorylation (Vilchinskaya et al., 2017) and, accordingly, reduced inhibitory effect of AMPK on mTORC1/p70S6K signaling pathway (Bolster et al., 2002). At the present study, we also observed a significant decrease in AMPK (Thr172) phosphorylation after 1-day HS, which was completely prevented by PMS.

### Catabolic Signaling Markers

It is known that mechanical unloading is accompanied by increased expression of muscle-specific E3 ubiquitin ligases MuRF-1 and MAFbx/atrogin-1 in rodent postural muscles both at the mRNA (Bodine et al., 2001; Wu et al., 2014; Baehr et al., 2017) and protein level (Wang et al., 2015; Belova et al., 2017; Ninfali et al., 2018). In the present study, we also found a significant increase in the mRNA expression of these E3 ubiquitin ligases. PMS prevented an increase in MuRF-1 expression but not in MAFbx/atrogin-1 expression. It appears that that there are distinct mechanisms involved in the regulation of these ubiquitin ligases, which was previously suggested by reports from our laboratory (Shenkman et al., 2015; Belova et al., 2017) as well as others (De Boer et al., 2007; Wu et al., 2014). MuRF-1 gene expression can be regulated through different pathways, including HDAC4/dach/MYOG, HDAC5/TFEB (Du Bois et al., 2015), p38MAPK (Derbre et al., 2012), NFκB (Jackman et al., 2013), and IRS1/Akt/FOXO3 (Glass, 2010; Liu et al., 2017). Each of these signaling pathways could potentially affect MuRF-1 mRNA during PMS. At the same time, PMS was not able to prevent the upregulation of MAFbx/atrogin-1 mRNA expression, which could possibly be associated with the failure of PMS to prevent a decrease in the phosphorylation level of both Akt and FOXO3. It is noteworthy that unloading-induced downregulation of PGC-1α expression, which is known to suppress FoxO-dependent transcription of ubiquitin ligases (Sandri et al., 2006), was partially attenuated by PMS. However, it appears that partial recovery of PGC-1α expression in the PMS group was insufficient to completely suppress the expression of atrogenes. The results of the present study also indicate that at the early stage of unloading (1 day) AMPK and PKD appear to be in reciprocal relations, i.e., a decrease in AMPK phosphorylation is accompanied by an increase in PKD phosphorylation, which leads to HDAC5 nuclear export and subsequent increase in MuRF-1 mRNA expression *via* PKD1/HDAC5/TFEB/MuRF1 pathway (Du Bois et al., 2015; Vilchinskaya et al., 2018). These effects were completely prevented by maintaining the tonic contractile activity of the soleus muscle *via* PMS. It is known that disuse conditions can induce an upregulation of the authophagy markers (Brocca et al., 2012; Egawa et al., 2015; Chibalin et al., 2018; Moller et al., 2019). The results of the present study have confirmed this notion, as the content of phospho-ULK1 (ser 555) in rat soleus muscle was upregulated following both 1-day and 3-day unloading.

## Conclusion

PMS for 4 h a day during 3-day hindlimb unloading can partially prevent a decrease in the global rate of protein synthesis in the rat soleus muscle. PMS during 3-day HS was able to completely prevent a decrease in GSK-3β phosphorylation as well as partly attenuate an increase in eEF2 phosphorylation. Thus at the early stage of unloading the mechanical stimulation of the support afferents maintains control phosphorylation levels of endogenous PS inhibitors (GSK3β and eEF2). However the significant reduction in the expression of the markers of ribosome biogenesis was not rescued by PMS. Complete prevention of MuRF-1 upregulation by PMS was accompanied by the maintenance of the “atrophic” character of Akt-signaling. Such a prevention of MuRF-1 expression may be explained by the reduced export of HDAC5 from the nucleus.

Several important signaling markers that significantly altered at the early stage of unloading did not respond to support stimulation. We may suggest two possible explanations for this phenomenon. These markers could be restored after longer period of PMS combined with the unloading when PMS effects might be accumulated. The second possibility is that the simulated background tonic activity of the muscle should be supplemented with a resistive component, which can be provided by axial loading on the soleus muscle.

## Data Availability Statement

The datasets generated for this study are available on request to the corresponding author.

## Ethics Statement

The animal study was reviewed and approved by Biomedicine Ethics Committee of the Institute of Biomedical Problems of the Russian Academy of Sciences/Physiology section of the Russian Bioethics Committee (protocol no. 414, 23.12.2015).

## Author Contributions

BS, EM, and ST designed the study. TM, ST, EM, SB, KS, SR, NV, and IP performed the experiments. ST, TM, and BS analyzed and interpreted the data and wrote the manuscript. All authors have approved the final version of the manuscript and agree to be accountable for all aspects of the work. All persons designated as authors qualify for authorship, and all those who qualify for authorship are listed.

### Conflict of Interest

The authors declare that the research was conducted in the absence of any commercial or financial relationships that could be construed as a potential conflict of interest.
